# Identification and Rational Design of a Novel Antibacterial Peptide Dermaseptin-AC from the Skin Secretion of the Red-Eyed Tree Frog *Agalychnis callidryas*

**DOI:** 10.3390/antibiotics9050243

**Published:** 2020-05-10

**Authors:** Zijian Gong, Xinjie Pei, Shen Ren, Xiaoling Chen, Lei Wang, Chengbang Ma, Xinping Xi, Tianbao Chen, Chris Shaw, Mei Zhou

**Affiliations:** 1 Natural Drug Discovery Group, School of Pharmacy, Queen’s University Belfast, Belfast BT9 7BL, Northern Ireland, UK; zgong05@qub.ac.uk (Z.G.); xpei01@qub.ac.uk (X.P.); c.ma@qub.ac.uk (C.M.); x.xi@qub.ac.uk (X.X.); t.chen@qub.ac.uk (T.C.); chris.shaw@qub.ac.uk (C.S.); m.zhou@qub.ac.uk (M.Z.); 2 College of Chinese Medicinal Materials, Jilin Agricultural University, Changchun 130118, China; rs0109@163.com

**Keywords:** antimicrobial peptide, frog skin secretion, dermaseptin, low resistance drug

## Abstract

Antibiotic resistance represents a tremendous contemporary clinical challenge. Given this challenge, antimicrobial peptides (AMPs) are regarded as one of the most promising new options for next-generation lead antibiotics. Here, we describe the antibacterial activities of a cationic peptide named DRP-AC4, obtained from frog skin secretion using shotgun cloning. Two modified peptides were derived by substituting the sequence of amino acids to complete the hydrophobic face (DRP-AC4b) and increase net charge (DRP-AC4a), respectively. The activity and cytotoxicity of these two peptides were compared. DRP-AC4a displayed significantly increased potency against bacteria compared to the natural peptide. It should be noted, however, that both analogue peptides demonstrated higher lytic ability than the natural peptide against the membranes of mammalian erythrocytes. At the same time, all three peptides displayed lower hemolytic activity compared to their antibacterial activity. Here, we demonstrate that AMPs have more complex activity mechanisms and faster bactericidal rates than traditional antibiotics, which may be one of the reasons why bacteria do not develop resistance to them. These discoveries provide interesting insights into the discovery and development of novel drugs from natural sources.

## 1. Introduction

Since Fleming’s discovery of penicillin in 1928, antibiotics have been widely used to treat infectious diseases in humans and animals for nearly 100 years [1]. Antibiotics have always played an essential role in protecting human health. However, due to the emergence of a large number of resistant bacteria, antibiotics have gradually lost their antibacterial utility [2,3]. Therefore, the development of new antibiotics or other antibacterial substances has become an urgent problem to be solved by the biomedical community [4]. Antimicrobial peptides (AMPs) were first studied in the 1980s [5]. AMPs are an indispensable and vital part of the innate immune systems of humans, animals and plants [6], and demonstrate a broad spectrum of activity and high potency against bacteria, fungi, and viruses [7]. Small changes in their amino acid sequences may lead to significant changes in AMP activity against different microorganisms [6]. Compared to traditional antibiotics, AMPs kill bacteria in a sophisticated manner and have a fast killing rate, thus, bacteria are not likely to develop resistance [8]. Therefore, these peptides could be used to treat a variety of diseases caused by microbes, and even those that are conventionally antibiotic-resistant.

The dermaseptins constitute a family of peptides with highly conserved sequences, usually of 28–34 amino acids [9]. Dermaseptins may have a variety of structures, but they also have typical conserved conformation motifs, including several lysine residues on the hydrophilic face of a helical structure and a tryptophan residue at the third position from the N-terminus in the mature peptide [10]. The tryptophan residue in the third position is a significant feature of dermaseptin family peptides that possess antibacterial functions, the possible reason being that the indole ring on the tryptophan side chain helps the peptide to form an α-helix structure when the peptide enters the cell membrane [11]. C-terminal amidation can increase the net positive charge of AMPs and facilitate their binding to negatively-charged cell membranes and avert peptide degradation in the natural environment [12]. Some dermaseptins have shown an effective, rapid and irreversible ability to inhibit microorganisms with low cytotoxicity to mammalian cells [13]. On the other hand, one regular feature of dermaseptin peptide action is interference with or disruption of the lipid bilayer of target cells [14].

Herein, we report the primary structure of a novel dermaseptin peptide named DRP-AC4, which was isolated from *Agalychnis callidryas* (*A. callidryas*) using shotgun cloning. The mature peptide was obtained for investigation by solid-phase synthesis, and bioactivity experiments showed that this peptide has a wide range of antimicrobial activities with a mechanism of action mainly through destruction of the cell membrane. In addition, two analogue peptides were designed by changing the type of amino acids in the sequence and the net charge in order to investigate the effects of hydrophobicity and net charge on the biological activity of the peptide. Furthermore, the results of drug resistance induction assays indicate that the sensitivity of bacteria to these AMPs did not decrease with long-term usage.

## 2. Results

### 2.1. Molecular Cloning of the Precusor Encoding a Novel Dermaseptin Peptide

**Figure 1:**
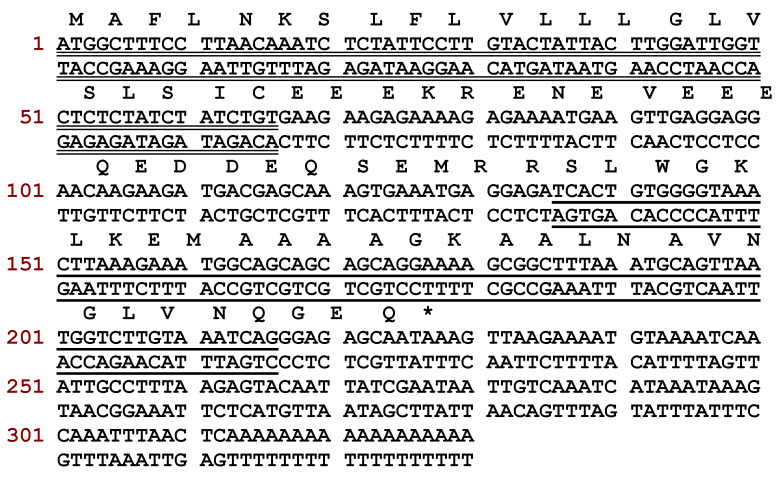
Nucleotide sequence of cloned cDNA encoding the biosynthetic precursor of DRP-AC4 from *A. callidryas* and translated amino acid sequence of the open reading frame. The putative signal peptide is double underlined, the mature peptide is single underlined and the stop codon is indicated by an asterisk.

**Figure 2:**
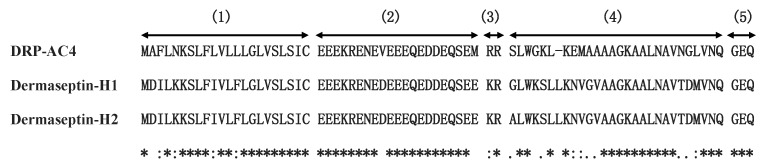
The alignment of full-length amino acid sequences and domain architecture of precursors encoding DRP-AC4, Dermaseptin-H1 and Dermaseptin-H2. (1) Signal peptide; (2) Acidic spacer peptide region; (3) Dibasic propeptide convertase processing site; (4) Mature peptide; (5) Glycine residue amide donor. Conserved amino acids are indicated by asterisks.

A peptide precursor encoding a putative novel peptide named DRP-4 was identified through translation of a cDNA, which was repeatedly cloned from a skin secretion-derived cDNA library of *A. callidryas* (Figure 1). The translated open-reading frame consisted of 75 amino acid residues containing a 22-residue signal peptide region, followed by a 21-residue Glu-rich acidic peptide spacer and a 27-residue mature peptide with a typical dermaseptin sequence released by cleavage after -RR- propeptide convertase processing. An extension peptide (-GEQ-) was also present in which the G residue acted as a donor for C-terminal amidation of the mature peptide. The alignment of DRP-AC4 with other dermaseptin peptides showed that all members share a highly conserved amino acid sequence (Figure 2). The new peptide, DRP-AC4, was thus classified as a member of the dermaseptin family. The nucleotide sequence of DRP-AC4 has been deposited in the GenBank database under the accession number MT153747.

### 2.2. Identification and Structural Characterisation of DRP-AC4

**Figure 3:**
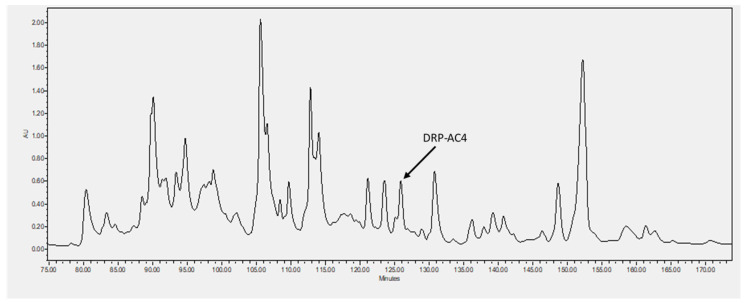
Reverse-phase HPLC chromatogram of Agalychnis callidryas skin secretion indicating elution/retention time of DRP-AC4 at 126 min (arrow). The Y-axis indicates absorbance units at λ = 214 nm.

**Figure 4:**
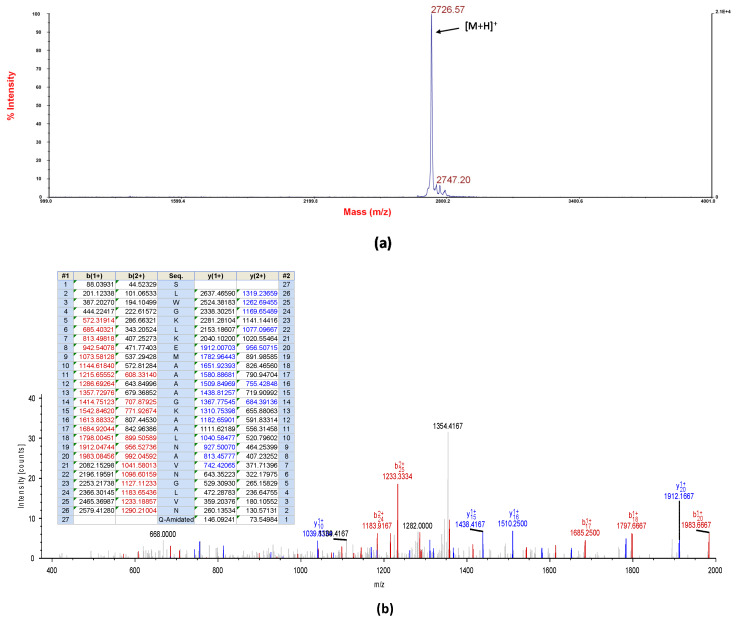
Mass spectra of DRP-AC4 isolated from frog skin secretion. (**a**) MALDI-TOF spectrum of the HPLC fraction at 126 min in Figure 3 corresponding to DRP-AC4; (**b**) Annotated MS/MS fragmentation spectrum. Predicted b- and y-ion MS/MS fragment ion series (singly- and doubly-charged) arising from MS/MS fragmentation. Observed ions are shown in coloured typeface.

The novel peptide, DRP-AC4, with a computed molecular mass of 2725.18 Da deduced from cloned skin cDNA, was identified in HPLC fraction with retention time at 126 min (Figure 3). The fraction containing masses coincident with the deduced putative peptide, which is analysed using MALDI-TOF mass spectrometry (Figure 4a), was subjected to MS/MS fragmentation sequencing, and the primary structure of DRP-AC4 was established (Figure 4b). 

### 2.3. Prediction of Secondary Structure and Structural Analysis of DRP-AC4 and Its Analogues

**Figure 5:**
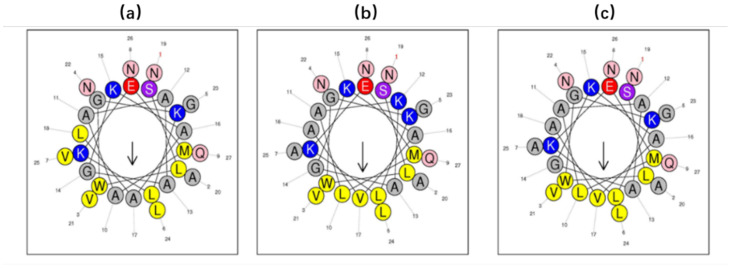
Predicted helical wheel projections of DRP-AC4 (**a**), DRP-AC4a (**b**) and DRP-AC4b (**c**).

Online analysis tools were used to predict the helical wheel plots and secondary structures. The parent peptide DRP-AC4 possessed a +3 net charge with a hydrophobicity of 0.341 and hydrophobic moment of 0.404 (Table 1). As can be seen in Figure 5, DRP-AC4b (LLVLWV) tended to have a more complete hydrophobic face than DRP-AC4 (LLAAWV), and although it has the same hydrophobicity as DRP-AC4, its hydrophobic moment was higher than DRP-AC4. Compared to DRP-AC4b, DRP-AC4a showed a lower hydrophobicity of 0.293 and had a single integer increase in positive charge on the hydrophilic face.

**Figure 6:**
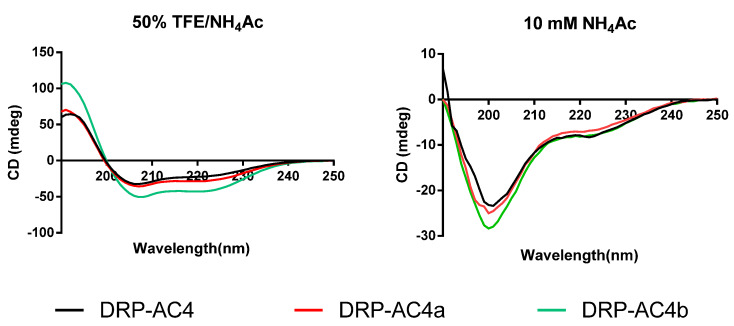
Circular dichroism spectra recorded for the three peptides (100 μM), DRP-AC4 (black), DRP-AC4a (red) and DRP-AC4b (green), in 50% TFE/NH_4_Ac (*v*/*v*) (**left**) and 10 mM NH_4_Ac buffer (**right**).

All synthesised peptides exhibited random coil structure in aqueous solution but folded into helical structure in a membrane-mimetic environment consisting of 50% trifluoroethanol (TFE) in 10mM ammonium acetate (NH_4_Ac) solution (Figure 6). The parent peptide and cationicity- and amphipathicity-enhanced analogue, DRP-AC4a, had a similar degree of conformational shift from random coil to helix. Optimising the amphiphilic structure with the same amino acid composition significantly increased the proportion of α-helix of the natural peptide in membrane-mimetic solution (Table 2). 

### 2.4. Antimicrobial Assay of DRP-AC4 and Its Analogues

The results for the minimum inhibitory concentrations (MICs) and minimum bactericidal concentrations (MBCs) of the three peptides are shown in Table 3, and they indicate moderate antibacterial activity. In addition to potent effects on *E. coli* and *S. aureus*, DRP-AC4a exhibited weaker inhibitory effects on *C. albicans* and *P. aeruginosa*. The MICs and MBCs of DRP-AC4a were generally reduced compared with DRP-AC4, while those of DRP-AC4 and DRP-AC4b were generally the same against most of the examined organisms. Interestingly, although the MICs of the three peptides on *S. aureus* and *E. coli* were the same, they had different MBCs. The geometric mean (GM) MICs for each peptide indicated that the antimicrobial activities of both designed analogues were increased, where the cationicity- and amphipathicity-enhanced analogue, DRP-AC4a, displayed the most potent inhibitory effects against the tested strains (Table 3 and Supplementary Materials, Figure S1).

### 2.5. Anti-Biofilm Activity

The three peptides had inhibitory effects on biofilm formation by *S. aureus* (Table 4 and Supplementary Materials, Figure S2). Similar to the antibacterial results, DRP-AC4a was more potent for anti-biofilm activity than DRP-AC4 and DRP-AC4b. However, the three peptides did not have significant eradication effects on the biofilms that had been formed.

### 2.6. Haemolytic Activity

**Figure 7:**
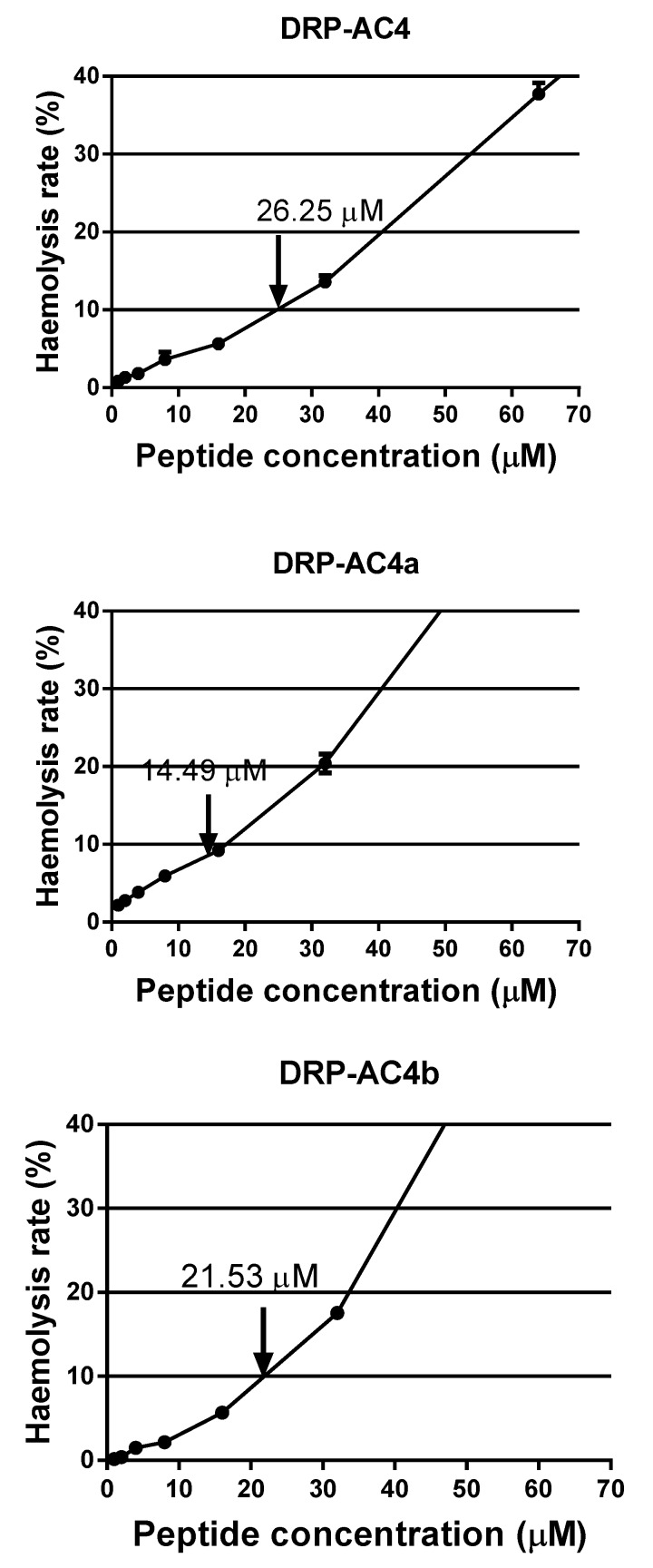
The haemolysis of horse red blood cells with different concentrations of DRP-AC4, DRP-AC4a and DRP-AC4b. The error bar represents the standard error of the mean (SEM) of fifteen replicates. Arrows indicate the haemolysis rate at the peptide concentration of the GM MICs.

As can be seen from the data in Figure 7, DRP-AC4, DRP-AC4a and DRP-AC4b were found to possess similar low levels of haemolytic activity (about 10%) at the MICs determined for seven different bacteria. In contrast, the haemolytic activity of DRP-AC4a at its GM MIC was slightly weaker than that of the other two peptides.

### 2.7. Permeabilisation Effects of Peptides on the Cell Membrane

**Figure 8:**
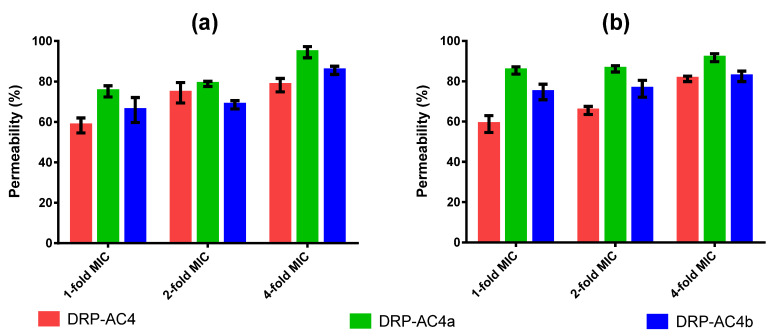
The destruction of *S. aureus* (**a**) and *C. albicans* (**b**) cell membranes at concentrations of 1-fold, 2-fold and 4-fold MICs. The error bar represents the SEM of fifteen replicates.

DRP-AC4 and its derivatives induced approximately 90% permeation of the cell membranes of *S. aureus* and *C. albicans* at 4 X MICs. DRP-AC4a exhibited a slightly higher penetration ability than the other two peptides (Figure 8). The permeabilisation effects of the three peptides on the cell membranes of the two microorganisms decreased with decreasing concentration. However, at the MICs of the peptides, at least 60% of the membranes were destroyed. 

### 2.8. Resistance Induction by Serial Passages in S. aureus

The serial passages of DRP-AC4 and the two analogues did not increase specific bacterial resistance (in Supplementary Materials, Figure S3). These results provided further support for the hypothesis that bacteria readily develop drug resistance to traditional antibiotics but not to AMPs, a phenomenon which may be related to the rapid sterilisation rate and more elaborate action mechanism of AMPs.

## 3. Discussion

An increasing number of research reports indicate that long-term use of conventional antibiotics can lead to microbial resistance. Many resistant bacteria have been identified that have been induced/selected by these traditional antibiotics [15]. In recent decades, increasing numbers of studies have been performed on AMPs. Through these studies, a plethora of bioactive natural peptides have been found, including anti-microbials, protease inhibitors, insulin secretion promoters, and so on. 

DRP-AC4 exhibited almost the same activity against Gram-positive and Gram-negative bacteria [16], although the cell walls of Gram-negative bacteria, composed of lipopolysaccharides, are thicker than those of the Gram-positive bacteria which consist of peptidoglycan. Bacterial membranes are mainly composed of lipid with amphiphilic properties (hydrophilic and hydrophobic), and not surprisingly, peptides must have hydrophobicity as it is a crucial factor for antibacterial activity. The hydrophobicity of DRP-AC4 and its analogues is between 0.293–0.341, which is in line with a range of AMPs with high antibacterial activity and low cytotoxicity. After the amino acids were modified in the sequence of DRP-AC4b to make the hydrophobic face more complete, the binding ability of the peptide to cancer cells became stronger than that of the parent peptide. It seems possible that these results are due to the completed hydrophobic face being more conducive to the formation of higher helicity. This assumption is also consistent with the CD results, where DRP-AC4a and DRP-AC4b possess a higher degree of helix structure in comparison of the natural peptide. 

The net positive charge carried by AMPs has been considered as being the critical factor in ensuring the electrostatic binding properties of AMPs to the negatively charged cell membrane. The lipopolysaccharides and phospholipids with a negative charge on the cell membrane bind to the AMPs and form voids, causing the leakage of cell materials [12]. Several reports have shown that there is a connection between net charge and potency with most active peptides possessing a number of net charges between +3 and +6; however, the optimum quantity has not yet been determined. This may be related to the type of amino acid, the length of the peptide chain and the secondary structure. Based on DRP-AC4b, DRP-AC4a replaced the non-charged alanine in the hydrophilic face with lysine, and the comprehensive MIC value for seven types of microorganism decreased from 21.53 μM to 14.49 μM. Initial observations suggested that positive charge plays a vital role in antibacterial activity, and lysine can enhance the binding ability of peptide to the bacterial membrane. However, the haemolytic activity and toxicity of DRP-AC4 were not improved. This suggests that improving the charge state promotes binding to the bacterial anionic membrane bilayer but does not affect the amphoteric bilayer in mammalian cells (erythrocytes and dermal microvascular endothelial cells) [17]. Therefore, when the hydrophobicity is controlled within a reasonable range, increasing the appropriate amount of positive charge can improve the antibacterial activity without causing cytotoxicity. 

In our previous study, it was reported that when dermaseptins are attached to the lipid bilayer, they can initiate an α-helical structural transformation, which is critical for antimicrobial activity [18]. It was noted here that the three peptides underwent a coil structure to helix structure transition in going from a water environment to a membrane mimic environment. Although using CD spectroscopy to calculate the helical content of a peptide may not be accurate, it can be seen from Figure 6 that DRP-AC4b possesses more helical content than DRP-AC4a in a membrane-mimetic environment. Thus, helicity content is not the only measure of antimicrobial activity.

Although DRP-AC4 has a broad spectrum of antibacterial activity, this does not mean that it is equally effective against biofilms, especially against established biofilms. DRP-AC4a at high concentrations eradicated biofilms formed by *S. aureus,* however, DRP-AC4 and DRP-AC4b had no effect on their eradication. This difference in activity may be due to changes in the number of charges as it was inferred that AMPs with multiple charges are more likely to destroy and degrade extracellular polymeric substances [19]. This seems to indicate that this peptide does not have the potential to become an anti-biofilm agent.

We studied the activity mechanism of three peptides and the experimental results indicated that the action of the three peptides on bacteria was mainly by destroying cell membranes (Figure 8), where the peptide dissolves the bilayer into a micellar structure that makes it unstable. Being cationic, they are capable of interacting with the negatively charged bacterial phospholipid heads, and are then inserted into the bilayer. It has been proposed that membrane dissolution occurs through numerous mechanisms including membrane perforation, solubilisation and disruption [20,21,22]. 

Antibiotics and AMPs differ not only in their antibacterial mechanisms but also in their killing speed. It is now understood that these two factors play an important role in why AMPs are less likely to induce resistance when compared to conventional antibiotics. In addition, AMPs can kill bacteria in 25 min at their MBCs, whereas in general, antibiotics take six to twelve hours to achieve the same effect [23]. In the mechanism of action, antibiotics usually act on a single molecular target to attack bacteria [24]. The relatively simple bactericidal mechanism and moderate bactericidal speed lead to a decrease or lack of bacterial sensitivity to antibiotics, resulting in these traditional antimicrobial agents ineffective. To study if this occurs with peptides, we performed resistance induction experiments. Our results revealed that the bactericidal activity of DRP-AC4 and its derivatives did not decrease after 16 cycles of induction at half the MIC. We can thus speculate that bacteria are unlikely to develop resistance to AMPs in a short time, although we cannot extrapolate these data to longer times or number of passages.

## 4. Materials and Methods

### 4.1. Skin Secretion Harvesting

Adult specimens of frogs were obtained from a commercial source in United Kingdom. The frogs were maintained in a specialised tropical frog facility on a 12-h/12-h day and night cycle in Queen’s University Belfast, and fed multivitamin-loaded crickets every two days for at least 4 weeks. The dorsal skin secretion of all specimens was obtained by electrical stimulation (6V DC; 4 ms pulse-width; 50 Hz) through platinum electrodes for two periods of 20s. The white skin secretions were rinsed of the skin with deionised water into a chilled beaker, snap-frozen in liquid nitrogen and lyophilised. The samples were stored at −20 °C before analysis. The study was performed according to the guidelines in the UK Animal (Scientific Procedures) Act 1986, project license PPL 2694, issued by the Department of Health, Social Services and Public Safety, Northern Ireland. Procedures were vetted by the Institutional Animal Care and Use Committee (IACUC) of Queen’s University Belfast and approved on 1 March, 2011.

### 4.2. Identification of AMP Precursor Encoding cDNAs from the Skin Secretion

Polyadenylated mRNA was isolated from the skin secretion using a Dynabeads mRNA Direct Kit (Invitrogen). The full-length sequences of the mRNA transcripts encoding the AMP precursors were captured using a SMART-RACE kit (Clontech U.K.) after reverse transcription. A nested universal primer (NUP) and a degenerate primer were employed in molecular cloning. Also, the degenerate primer (5′-ACTTTCYGAWTTRYAAGMCCAAABATG-3′) was designed to a highly conserved domain of the 5′-UTR of previously characterised homologous AMP precursor-encoding cDNAs from *Phyllomedusa* species. The polymerase chain reaction (PCR) cycling procedure was carried out as follows: initial denaturation step for 60 s at 94 °C, 40 cycles of denaturation for 30 s at 94 °C, primer annealing for 30 s at 60 °C; extension for 180 s at 72 °C. PCR products were subjected to purification and cloning by use of a pGEM-T vector system (Promega, USA), and then sequenced using an ABI 3100 Automated Capillary Sequencer (Biosystems, USA).

### 4.3. Identification and Structural Characterisation of the Predicted Peptide DRP-AC4 from the Skin Secretion of A. callidryas

Five milligrams of lyophilised *A. callidryas* skin secretion were dissolved in 1ml of water/trifluoracetic acid (TFA) (99.95/0.05, v/v) and clarified of microparticulates by centrifugation. The supernatant was pumped directly into a reverse-phase HPLC system (Waters, Milford, MA, USA) fitted with an analytical column (Phenomenex C-18, 25 cm× 0.45 cm Macclesfield, Cheshire, UK). Peptides were eluted with a linear gradient mobile phase from water/trifluoracetic acid (TFA) (99.95/0.05, v/v) to acetonitrile/water/TFA (80.00/19.95/0.05, v/v) at a flow rate of 1ml/min for 240 min. The fractions (1ml) were collected each minute and then analysed using MALDI-TOF MS (matrix assisted laser dissociation ionised-time of flight mass spectrometry) (Voyager DE, Perspective Biosystem, Foster City, CA, USA) in positive detection mode using CHCA (α-cyano-4-hydroxycinnamic acid) as matrix. Then, fractions containing masses coincident with the deduced putative novel cDNA-encoded peptide were injected into an LCQ-Fleet electrospray ion-trap mass spectrometer (Thermo Fisher Scientific, San Francisco, CA, USA) and sequenced using MS/MS fragmentation sequencing.

### 4.4. Peptide Design and Synthesis

The sequence of the natural peptide (DRP-AC4) was established as SLWGKLKEMAAAAGKAALNAVNGLVNQ-NH2, and it was utilised as a template to design two variants, including DRP-AC4a and DRP-AC4b, to improve the microbiocidal activity of the peptide. Residues at position 10, 12, 17, 18 and 25 of DRP-AC4 were replaced by leucine, lysine, valine, alanine and alanine, to construct a new analogue termed DRP-AC4a with enhanced cationicity and amphipathicity. Another analogue, DRP-AC4b, involved favouring an amphipathic structure with the same composition of the parent peptide but with the amino acids in a slightly different order, where A^10^, A^17^, L^18^ and V^25^ were substituted with leucine, valine, alanine and alanine, respectively. The three peptides were synthesised using a Tribute peptide synthesiser (Protein Technologies, Tucson, AZ, USA). A mixed solution of trifluoroacetic acid (TFA), ethanedithiol (EDT), triisopropyl silane (TIS) and water (94:2:2:2 (v/v)) was used to cleave the products for deprotection and separated from the resin. The products were purified by reverse-phase HPLC (Phenomenex C-5 column, 0.46 cm*25 cm) and masses of synthesised products in each case were verified by LCQ-Fleet electrospray ion-trap mass spectrometry (Thermo Fisher Scientific, San Francisco, CA, USA) (The HPLC chromatograms of purified peptides and corresponding mass spectra were provided in in Figures S4–S6) 

### 4.5. Physical and Chemical Property Predictions, Peptide Secondary Structure Predictions and CD Analyses

The Heliquest webserver (https://heliquest.ipmc.cnrs.fr/) was used to predict the physicochemical properties of the three peptides and helical wheel plots of the secondary structures were obtained by constructing helical wheels [25]. Moreover, CD analyses were carried out on a JASCO J815 Spectropolarimeter (JASCO Inc., Easton, MD, USA). The measuring range of samples was 190–250 nm, the peptide samples (100 µM) were prepared in 10mM NH_4_Ac solution and 50% TFE/NH_4_Ac (v/v), respectively. The BESTSEL online software (http://bestsel.elte.hu/index.php) was applied to predict the percentage of the α-helix structure.

### 4.6. MIC and MBC Assays

Seven different bacteria were used to determine the MICs and MBCs for the peptides. The Gram-positive bacteria were *S. aureus* (NCTC 10788), methicillin-resistant *S. aureus* (MRSA) (NCTC 12493) and *E. faecalis* (NCTC 12697) and the Gram-negative bacteria were *P. aeruginosa* (ATCC 27853), *E. coli* (NCTC 10418) and *K. pneumoniae* (ATCC 43816). The yeast employed was *C. albicans* (NCTC 1467). All microorganisms were cultured in Mueller-Hinton broth (MHB) to log phase growth and reconstituted to 5 × 10^5^ colony forming units (CFU)/ml. The peptide solutions and norfloxacin solutions, in the final concentration range of 1–512 μM (in two-fold dilutions), were added to the bacterial suspensions in a 96-well plate and incubated at 37 °C for 16–24 h. A no drug group was used as a growth control and norfloxacin was used as a positive control. After incubation, the Synergy HT plate reader (Biotech, USA) was used to determine the optical density (OD) values at 550 nm of each well. MIC represents the lowest concentration of peptides without bacterial growth. Furthermore, 10 µl of bacterial suspensions from each well were inoculated onto Mueller-Hinton agar (MHA) plates. After 24 h, the concentration of the peptide that did not produce any bacterial growth was deemed to be the MBC. The GM MICs for the three peptides to inhibit the growth of the seven tested bacteria, was calculated to estimate the overall antimicrobial activity of the peptides.

### 4.7. Biofilm Assays

To test the inhibitory and eradicative effects of the three peptides on bacterial biofilms, *S. aureus* (NCTC 10788) was selected for MBIC and MBEC assays. Bacteria were cultured in Tryptic Soy Broth (TSB). The final concentration of peptide solutions were from 1–256 μM (in two-fold dilutions) and each bacterial culture at 10^6^ CFU/ml was incubated at 37 °C in the medium with shaking (200 rpm) in a 96-well plate (100 μL/well). After incubating for 18 h, deionised water was used to rinse every well to remove the planktonic bacteria. Then, 125 μL of methanol was used for immobilisation of the biofilm. After 10 min, the methanol was removed, the wells were dried and stained with 125 μL of 0.1% crystal violet for 30 min. Excess stain was removed by rinsing with deionised water. After drying, stained biofilm in each well was dissolved using 150 μL of 30% glacial acetic acid. When the biofilms in all the wells were dissolved, the glacial acetic acid was transferred to a new 96-well plate, and the absorbance at 595 nm was determined by the Synergy HT plate reader.

### 4.8. Haemolysis Assays

The haemolysis assay was used to detect the dissolution and rupture of the red blood cells by the AMPs. After the fresh horse erythrocytes (TCS Biosciences Ltd. Buckingham, UK) were washed by PBS, the volume of horse erythrocytes in suspension was fixed to 2% with PBS. Peptides with final concentrations of 1–512 μM, were added to the uniform suspensions of horse erythrocytes. Negative and positive controls were compared with PBS and 1% Triton x-100, respectively. After incubation for 2 h at 37 °C, samples were centrifuged at 1000× *g* for 5 min. The supernatant from each tube was transferred to a 96-well plate, and the OD values were determined using a Synergy HT plate reader at 550 nm.

### 4.9. Bacterial Cell Membrane Permeability Assays

SYTOX Green Nucleic Acid Stain (Life Technologies, UK) quickly penetrates cells with compromised plasma membranes but does not cross the membranes of live cells, making it a useful indicator of dead cells within a population. *S. aureus* and *C. albicans* were cultured in TSB. After that, 5% TSB in 0.85% sodium chloride solution was used to wash and resuspend the microorganisms. Fifty microlitres of bacterial suspension (1 × 10^8^ CFU/ml) was incubated with peptide solution in final concentrations of 1*MIC, 2*MIC and 4*MIC in a black 96-well plate (Sterilin, UK) at 37 °C for 2 h. Seventy percent isopropanol and fresh 5% TSB were used to process the same amount of microorganisms as in positive control and negative control, respectively. Two hours later, SYTOX Green at a final concentration of 5 μM was added to each well and shaken incubated for 5 min at 37 °C. Finally, the fluorescent intensity at 485 nm for excitation and 528 nm for emission were measured by the Synergy HT plate reader.

### 4.10. Resistance Induction by Serial Passages

Based on the methods of others [26], a drug resistance induction test was employed. Three peptides were tested for antimicrobial activity. One half of the MIC bacterial suspension was taken and transferred to fresh MHB. Then the culture was continued, the MIC assay was repeated, and this process was repeated for 16 cycles.

### 4.11. Statistical Analysis

All the results were obtained from at least three replicates of experiments. Data were analysed using GraphPad Prism 6 software. The SEM was used to represent the error bars in the graphs.

## 5. Conclusions

In conclusion, we have shown here that selective modifications of a natural AMP can alter its bioactivity in a positive manner, which makes such peptides more attractive as leads for new generations of antibiotics with fewer of the resistance-inducing properties of conventional antibiotics. Two peptide analogues, DRP-AC4a with increased cationicity and amphipathicity, and DRP-AC4b with an optimised amphipathic structure with the same composition of the parent peptide were designed to improve the microbiocidal activity of the natural peptide. As expected, both analogues possessed more helix structure and increased antimicrobial activity compared to the parent peptide. 

## Figures and Tables

**Table 1:** Physiochemical properties of DRP-AC4, DRP-AC4a and DRP-AC4b predicted by the Heliquest online website.

Peptides	Length (aa)	Molecular Weight (g/mol)	Net Charge at PH7	Hydrophobicity (<H>)	Hydrophobic Moment (<µH>)
DRP-AC4	27	2725.18	3	0.341	0.404
DRP-AC4a	27	2782.27	4	0.293	0.526
DRP-AC4b	27	2725.18	3	0.341	0.492

**Table 2:** Amino acid sequences and calculated of α-helix proportion of DRP-AC4, DRP-AC4a and DRP-AC4b.

Peptides	Amino Acid Sequence	% of α-Helix in 50% TFE/(*v*/*v*) ^a^
DRP-AC4	SLWGKLKEMAAAAGKAALNAVNGLVNQ-NH_2_	22.4
DRP-AC4a	SLWGKLKEMLAKAGKAVANAVNGLANQ-NH_2_	26.9
DRP-AC4b	SLWGKLKEMLAAAGKAVANAVNGLANQ-NH_2_	38.8

a—the % of α-helix in 50% TFE (v/v), which was calculated from the spectra in Figure 6 using BESTSEL online software. Different amino acid residues in comparison to the parent peptide are highlighted in red.

**Table 3:** Inhibitory and bactericidal effects of peptides on different microorganisms.

MIC/MBC (μM)			
Microorganisms	DRP-AC4	DRP-AC4a	DRP-AC4b
*S. aureus*	8/32	8/16	8/32
*E. coli*	8/8	8/8	8/16
*C. albicans*	64/128	16/32	64/128
*P. aeruginosa*	64/128	32/128	32/128
*E. faecalis*	32/64	8/32	32/32
*K. pneumoniae*	32/32	8/16	32/64
MRSA	32/64	8/32	16/32
GM	26.25	14.49	21.53

**Table 4:** Inhibitory and eradicative activities of DRP-AC4, DRP-AC4a, and DRP-AC4b against *S. aureus* biofilms. MBIC stands for minimum biofilm inhibitory concentration and MBEC means minimum biofilm eradication concentration.

MBIC/MBEC (μM)			
Microorganisms	DRP-AC4	DRP-AC4a	DRP-AC4b
*S. aureus*	32/>256	16/64	32/256

## Supplementary Materials

The following are available online at https://www.mdpi.com/2079-6382/9/5/243/s1, Figure S1: Inhibitory effects of DRP-AC4, DRP-AC4a and DRP-AC4b against (a) *S. aureus*, (b) *E. coli*, (c) *C. albicans*, (d) *P. aeruginosa*, (e) *E. faecalis*, (f) *K. pneumoniae* and (g) MRSA in a range of concentrations from 512 μM to 1 μM. Figure S2: Inhibition effects of DRP-AC4 (red), DRP-AC4a (green) and DRP-AC4b (blue) against the biofilm formed by *S. aureus*. Figure S3: Assessment of resistant induction of DRP-AC4 (red), DRP-AC4a (green) and DRP-AC4b (blue) in *S. aureus* after 16 passages. Figure S4: Reverse-phase chromatogram (a) and full scan mass spectrum (b) of purified DRP-AC4. Figure S5: Reverse-phase HPLC chromatogram (a) and full scan mass spectrum (b) of purified DRP-AC4a. Figure S6: Reverse-phase HPLC chromatogram (a) and full scan mass spectrum (b) of purified DRP-AC4b.
