# Supplementary material for: Identification and Rational Design of a Novel Antibacterial Peptide Dermaseptin-AC from the Skin Secretion of the Red-Eyed Tree Frog Agalychnis callidryas
Source: Antibiotics (Basel). 2020 May 10;9(5):243. doi: 10.3390/antibiotics9050243 (PMC7277532; doi:10.3390/antibiotics9050243)
Supplement: Supplementary file 1 [file antibiotics-09-00243-s001.pdf]

Identification and rational design of a novel antibacterial peptide dermaseptin-AC from the skin secretion of the red-eyed tree frog *Agalychnis callidryas*

Supplement materials

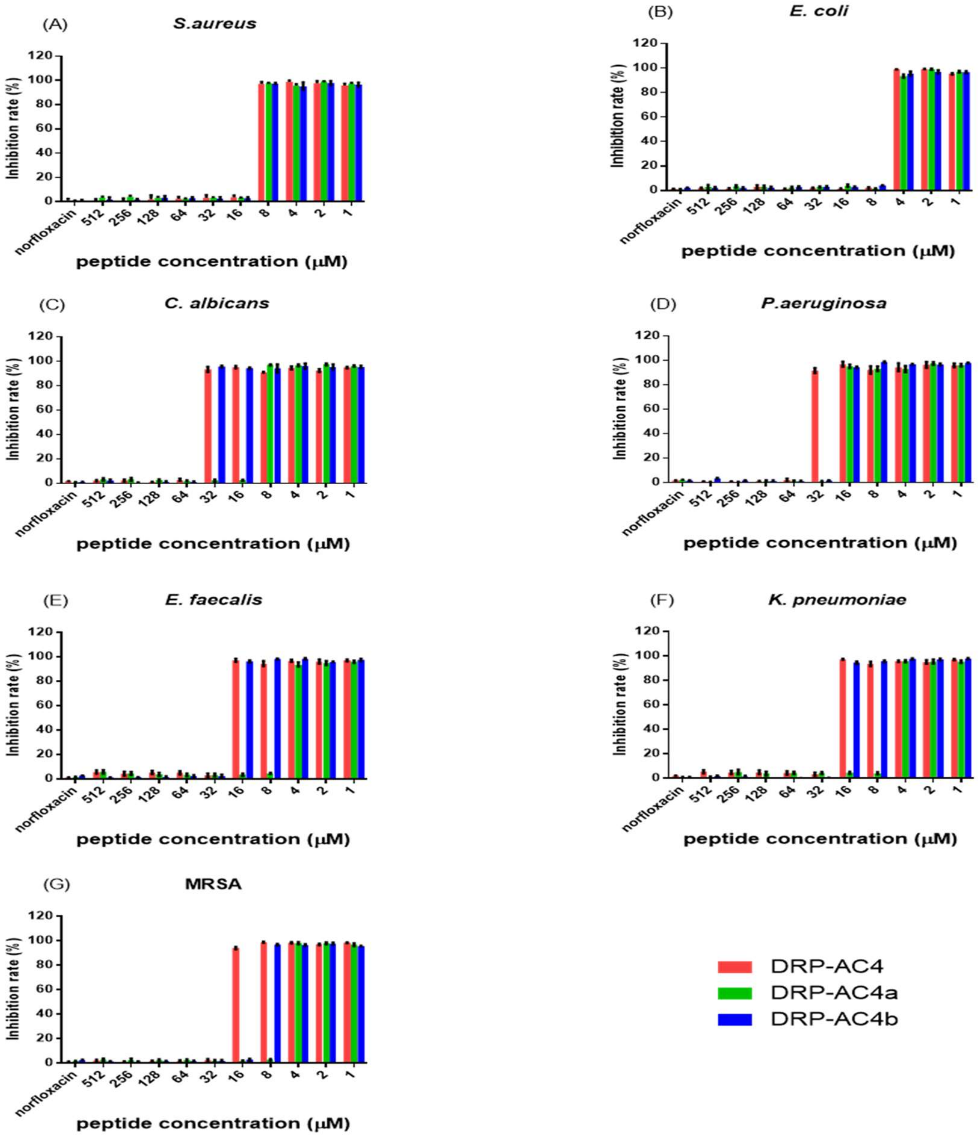

Figure S1 Inhibitory effects of DRP-AC4, DRP-AC4a and DRP-AC4b against (a) *S. aureus*, (b) *E. coli*, (c) *C. albicans*, (d) *P. aeruginosa*, (e) *E. faecalis*, (f) *K. pneumoniae* and (g) MRSA in a range of concentrations from 512 μM to 1 μM. Data represent means ± SEM.

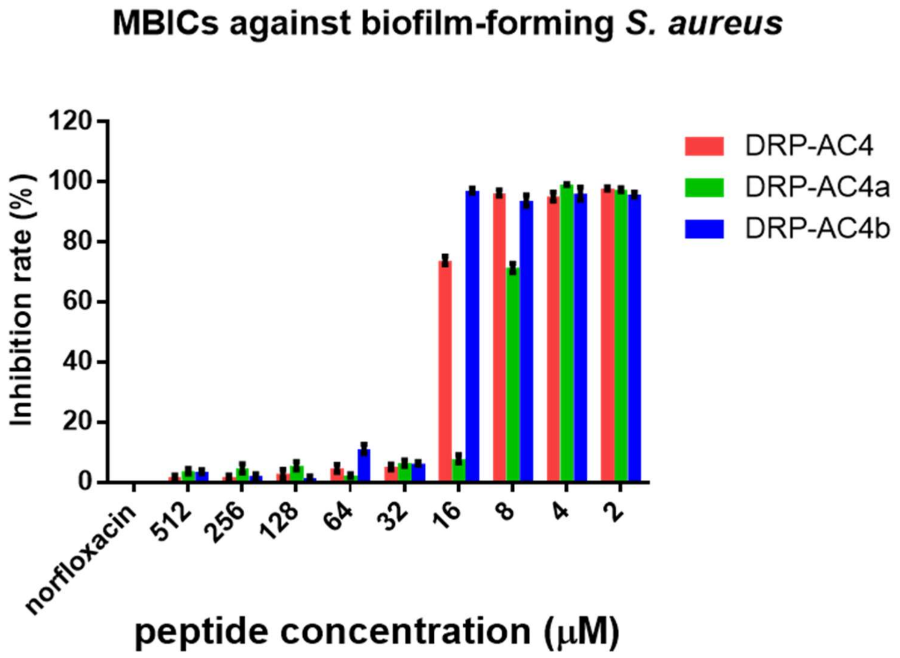

Figure S2 Inhibition effects of DRP-AC4 (red), DRP-AC4a (green) and DRP-AC4b (blue) against the biofilm formed by *S. aureus*. Data represent means ± SEM.

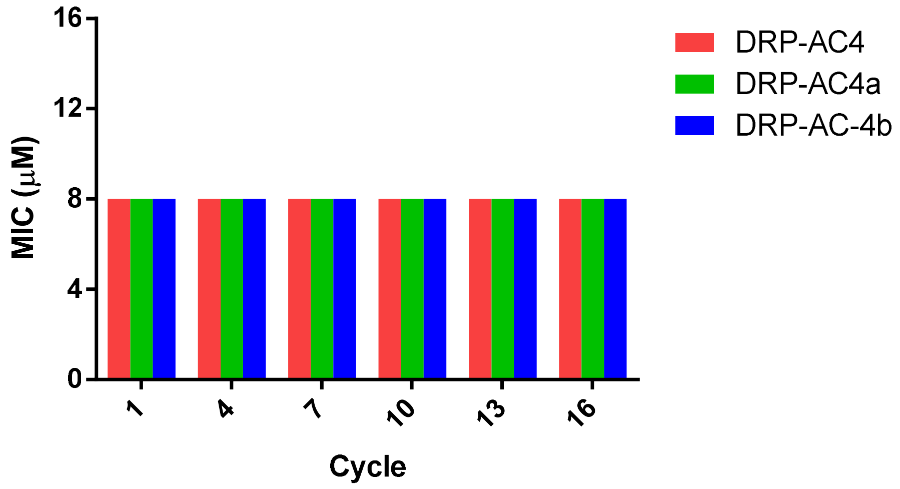

Figure S3 Assessment of resistant induction of DRP-AC4 (red), DRP-AC4a (green) and DRP-AC4b (blue) in *S. aureus* after 16 passages. The 1/2*MIC bacterial suspension was further cultured after antibacterial assay. The vertical axis represented MIC data and the horizontal axis represented the number of passages.

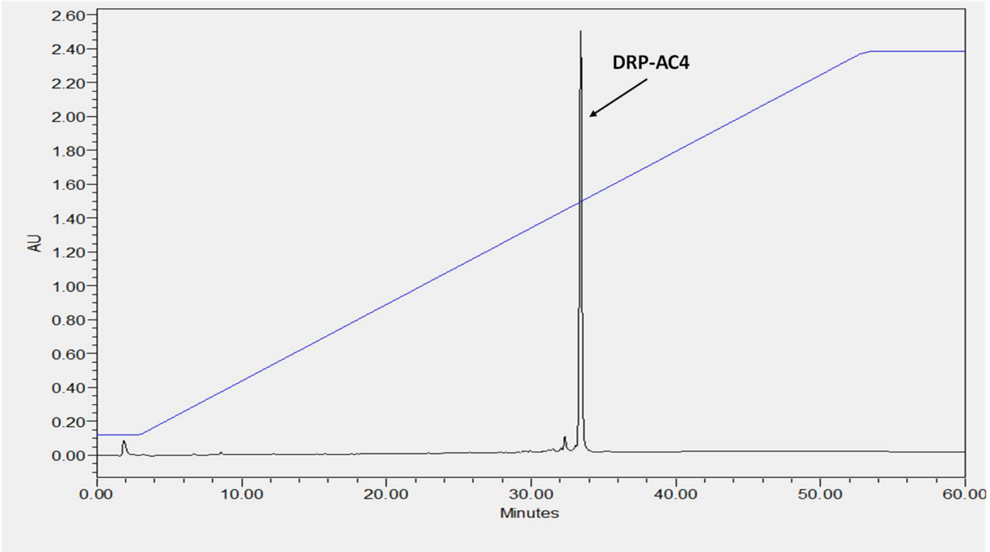

(a)

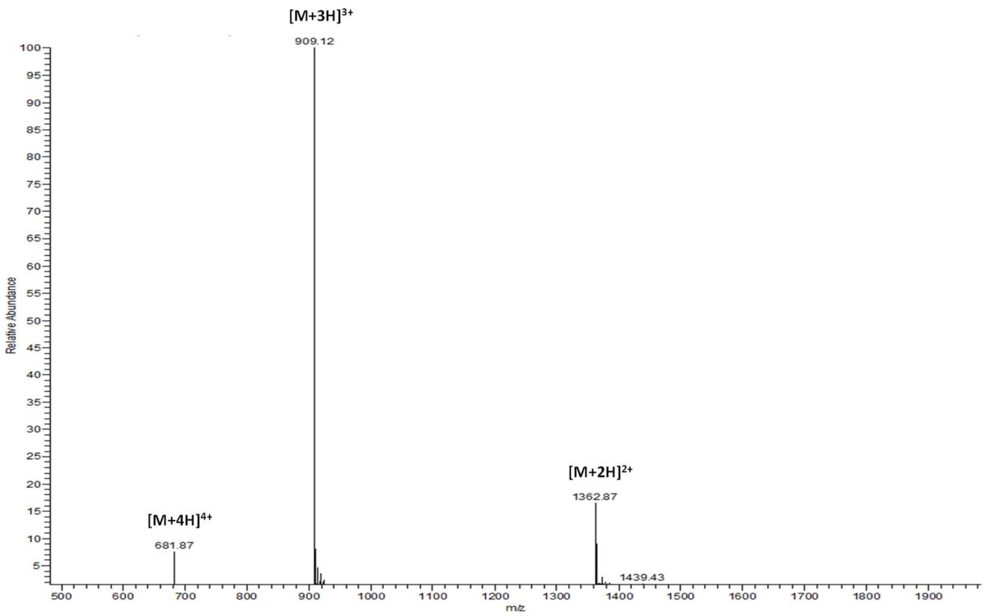

(b)

Figure S4 Reverse-phase HPLC chromatogram (a) and full scan mass spectrum (b) of purified DRP-AC4. The acetonitrile gradient is indicated by solid line. Multiple charged ions: $[M+2H]^{2+}$, $[M+3H]^{3+}$ and $[M+4H]^{4+}$.

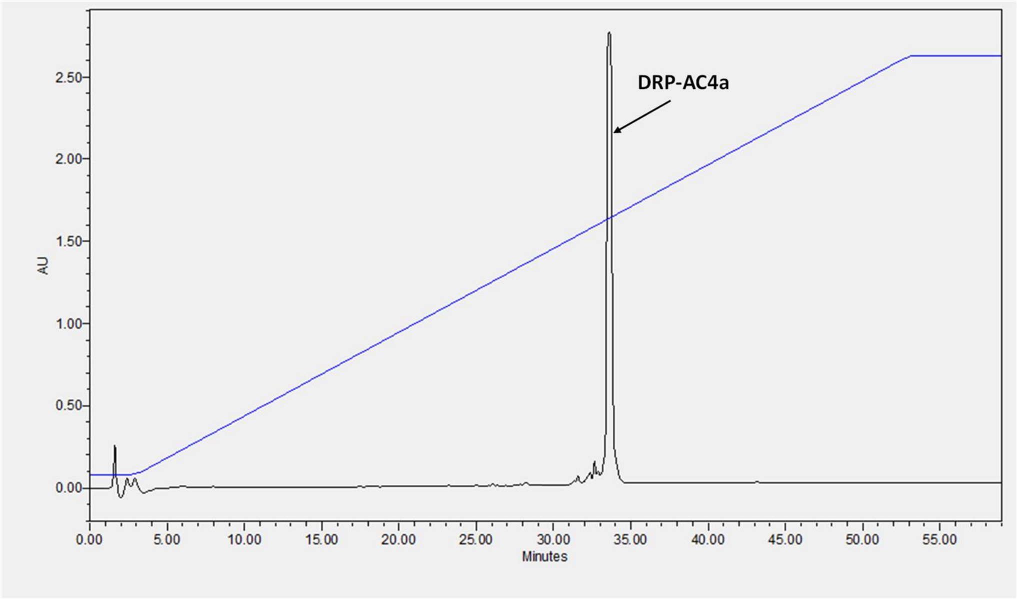

(a)

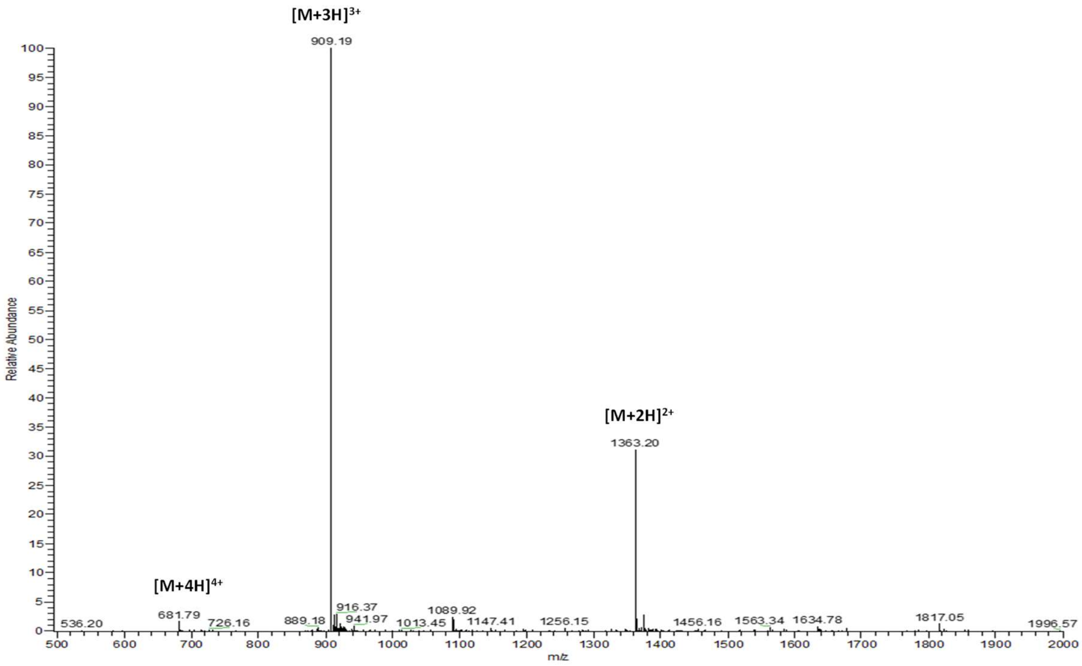

(b)

Figure S5 Reverse-phase HPLC chromatogram (a) and full scan mass spectrum (b) of purified DRP-AC4a. The acetonitrile gradient is indicated by solid line. Multiple charged ions: $[M+2H]^{2+}$, $[M+3H]^{3+}$ and $[M+4H]^{4+}$.

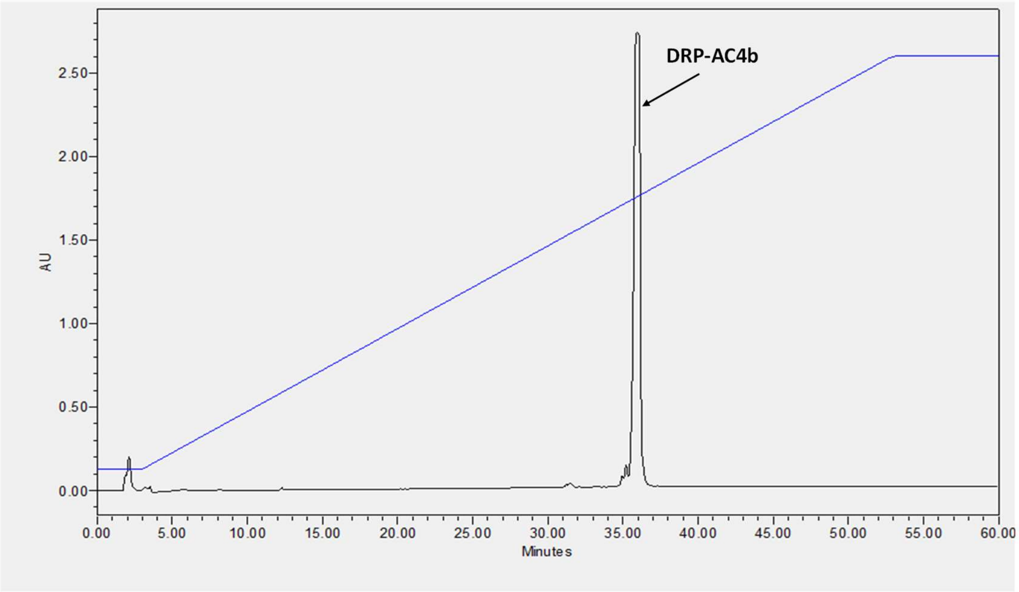

(a)

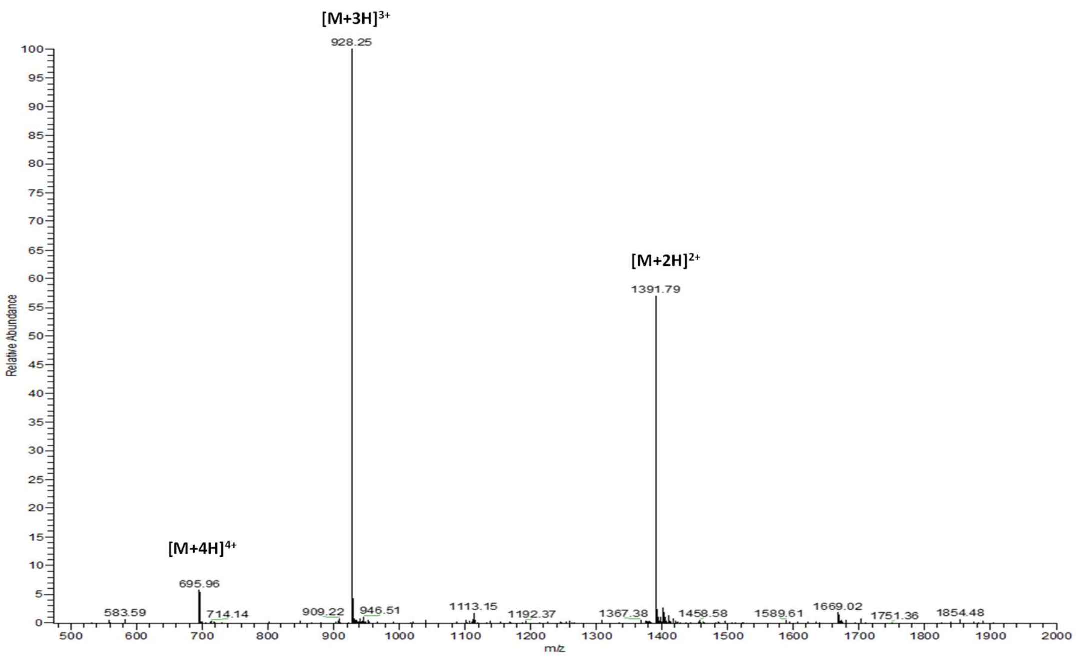

(b)

Figure S6 Reverse-phase HPLC chromatogram (a) and full scan mass spectrum (b) of purified DRP-AC4b. The acetonitrile gradient is indicated by solid line. Multiple charged ions: $[M+2H]^{2+}$, $[M+3H]^{3+}$ and $[M+4H]^{4+}$.
